# Virtual Reality-Assisted Rehabilitation for Upper-Limb Function in Stroke Survivors: A Systematic Review

**DOI:** 10.3390/brainsci16040417

**Published:** 2026-04-16

**Authors:** Ruxandra Pop-Kun, Anamaria Truță, Emanuel Ștefănescu, Dafin Mureșanu, Ștefan Strilciuc, Simona Clichici

**Affiliations:** 1 Physiology Department, “Iuliu Hațieganu” University of Medicine and Pharmacy, 400347 Cluj-Napoca, Romania; pop.kun.ruxandra.claudia@elearn.umfcluj.ro (R.P.-K.), sclichici@umfcluj.ro (S.C.); 2 Department of Genomics, MEDFUTURE Institute for Biomedical Research, “Iuliu Hațieganu” University of Medicine and Pharmacy, 400337 Cluj-Napoca, Romania; anamaria.truta@umfcluj.ro (A.T.); stefan.strilciuc@brainscience.ro (Ș.S.); 3 RoNeuro Institute for Neurological Research and Diagnostic, 400364 Cluj-Napoca, Romania; emanuel.stefanescu@brainscience.ro; 4 Department of Clinical Neurosciences, “Iuliu Hatieganu” University of Medicine and Pharmacy, 400012 Cluj-Napoca, Romania

**Keywords:** virtual reality, rehabilitation, upper limb

## Abstract

**Background:** Upper-limb impairment is a major contributor to chronic disability after stroke. Conventional recovery protocols frequently suffer from poor adherence, limited accessibility, and insufficient intensity for prolonged rehabilitation. **Methods:** We performed a systematic analysis of randomized controlled trials (RCTs) and non-randomized designs published between 2019 and 2024, assessing virtual reality (VR) interventions for upper-limb stroke rehabilitation. Participant characteristics, VR intervention details, primary and secondary outcomes, and adherence rates were analyzed in accordance with PRISMA guidelines. The review is registered in PROSPERO (CRD420251150877). We searched PubMed, Embase, Wiley, Scopus, and Cochrane databases. Study quality was assessed using the RoB 2 and ROBINS-I tools. This review received no funding. **Results:** Forty-one trials met the inclusion criteria. High variability in study methodology, VR devices, intervention protocols, and outcome measures limited direct comparability. Dropout rates were low and were frequently attributed to factors unrelated to the VR intervention. Adverse events were uncommon, supporting the feasibility and safety of VR-based rehabilitation. **Conclusions:** While VR is a safe and feasible modality, large-scale, multicenter clinical trials with standardized protocols and long-term follow-up are essential to define the role of VR in routine stroke care.

## 1. Introduction

Stroke is a major public health concern due to its increasing incidence, the long-term disability rate characterized by persistent upper-limb impairments in a substantial proportion of stroke survivors, and the poor quality of life (QoL) among those affected [1,2,3,4,5,6,7,8]. Conventional neurorehabilitation protocols often face challenges related to patient engagement, compliance, motivation, and long-term accessibility, highlighting the need for innovative neurorehabilitation strategies that ensure long-term accessibility, motivation, and a higher adherence rate to prolonged stroke rehabilitation protocols [9,10,11,12,13].

Despite promising preliminary findings, the heterogeneity of proposed VR neurorehabilitation systems, protocols, and devices underscores the challenges in evaluating their efficacy and clinical implementation. Our analysis aims to evaluate the effectiveness of VR-based interventions in upper-limb rehabilitation post-stroke. The key challenges associated with conventional rehabilitation protocols and the potential benefits of VR-based interventions are summarized in Table 1.

This systematic review summarizes findings from RCTs and non-randomized designs, including observational studies, single-case reports, and cross-sectional designs. We examined patient populations, device characteristics, intervention protocols, clinical implementation parameters, selected outcomes, and reported dropout rates (including reasons for attrition) to inform the future integration of VR-based systems into routine clinical rehabilitation practice. Sustained rehabilitation requires long-term accessibility and higher adherence rates to prolonged stroke rehabilitation protocols; however, current evidence regarding these long-term participation outcomes remains limited and inconsistent.

## 2. Materials and Methods

### 2.1. Registration and Search Strategy

This systematic review (SR) was conducted according to PRISMA 2020 guidelines to provide methodological accuracy and reproducibility. The protocol was developed a priori by defining research questions, eligibility criteria, search strategy, data extraction and analysis approach. The present protocol was registered in PROSPERO (CRD420251150877); a link is provided in Supplementary Material S1.

A comprehensive literature search was conducted in five electronic databases: PubMed, Embase, Wiley Online Library, Scopus, and the Cochrane Central Register of Controlled Trials (CENTRAL). The search was limited to studies published between 1 January 2019 and 4 December 2024 to capture the most recent advances in VR-based stroke rehabilitation. This window was chosen to ensure the inclusion of trials utilizing contemporary VR technology (e.g., consumer-grade HMDs, BCI integration, and advanced motion sensors). Studies prior to 2019 often relied on obsolete hardware and non-standardized software architectures that do not reflect current clinical rehabilitation practices. Search combined controlled vocabulary (e.g., MeSH, Emtree) and free-text terms for stroke, upper limb, virtual reality, and rehabilitation, with Boolean operators and filters for RCTs and controlled clinical trials where applicable. Only studies published in English and involving human participants were considered.

All search strategies are provided in Supplementary Material S2.

Eligibility criteria for study inclusion are summarized in Table 2.

### 2.2. Data Extraction and Quality Assessment

All references retrieved from the database searches were imported into Zotero for reference management and duplicate removal. Titles, abstracts, and full texts were screened for eligibility in accordance with the predefined inclusion and exclusion criteria. Screening, eligibility assessment, and data extraction were conducted using Microsoft Excel. Discrepancies between reviewers were resolved through discussion until consensus was reached.

Data extraction was performed independently by two reviewers using a standardized, pilot-tested form.

Extracted variables included study design and methodological quality (randomization, blinding, statistical methods applied); participant characteristics (demographic data, stroke type and stage, baseline upper-limb status); intervention characteristics (VR device and format—immersive, non-immersive, hybrid—session frequency, intensity, duration, and therapist involvement); and outcomes, with primary outcomes focusing on motor and functional recovery measured by the FMA or equivalent tools, and secondary outcomes covering dexterity, motor function, spasticity, functional independence, cognition, mood, and QoL, with the corresponding assessment instruments. Adherence and safety data (dropout rates with reasons for withdrawal and reported adverse events) were also collected.

All extracted data were cross-checked for accuracy, and discrepancies were resolved by consensus.

Quantitative data were tabulated to allow cross-study comparisons, while qualitative aspects were clustered according to stroke stage and VR modality (immersive vs. non-immersive).

Risk of bias was assessed using the RoB 2 tool for randomized controlled trials and the ROBINS-I tool for non-randomized studies (including observational, single-case and cross-sectional). The results were integrated to highlight methodological strengths, limitations, and evidence gaps across the included studies. Initial assessments were conducted by one reviewer and subsequently independently verified by a second senior reviewer (S.S.). Discrepancies in risk judgments were resolved through iterative discussion until a consensus was reached, adhering to the dual-review recommendations of the Cochrane Handbook and PRISMA 2020 guidelines.

Due to substantial heterogeneity in study designs, VR intervention protocols, devices used, outcome measures, and assessment time points, pooling of data for meta-analysis was not feasible. Instead, findings are presented as a narrative synthesis.

This systematic review was conducted in accordance with the PRISMA 2020 guidelines. The completed PRISMA 2020 checklist detailing the reporting of this study is provided in Supplementary Material Table S1, and the specific PRISMA checklist for the abstract is provided in Supplementary Material Table S2.

## 3. Results

### 3.1. Study Selection

**Figure 1:**
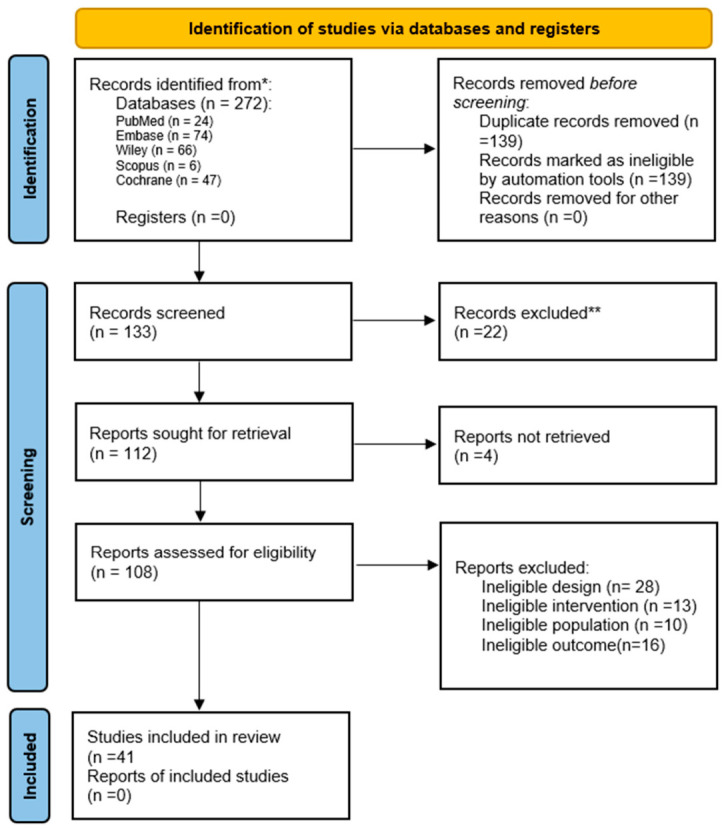
PRISMA flow diagram of study selection for the systematic review. * Records identified are reported per database, ** All screening assessments were conducted manually without the use of automation tools.

The initial database search identified 272 records. After the removal of 139 duplicates, 133 unique records were screened by title and abstract. Of these, 92 studies were excluded for not meeting the predefined eligibility criteria, most commonly due to non-stroke populations, non-VR interventions, lack of upper-limb outcomes, or publication type (reviews, protocols, or conference abstracts). Four reports were not retrievable despite efforts to obtain the full texts via institutional subscriptions and university library services. As eligibility could not be determined, these records were excluded from the review. The titles of studies excluded after full-text review *(n* = 67), along with the primary reasons for exclusion, are provided in Supplementary Material Table S3 to enhance transparency. The remaining 41 studies were assessed in full text and all were deemed eligible for inclusion in this review. The selection process is summarized in Figure 1 (PRISMA flow diagram). The review protocol was developed a priori, defining the research question, search strategy, data extraction, and analysis procedures. A comprehensive summary of the main results of all included studies is provided in Supplementary Material Table S4 to facilitate comparison and interpretation.

### 3.2. Study Characteristics

Among the 41 included trials, 38 (92.6%) were RCTs, with only one cross-sectional, one observational, and one single-case study.

**Figure 2:**
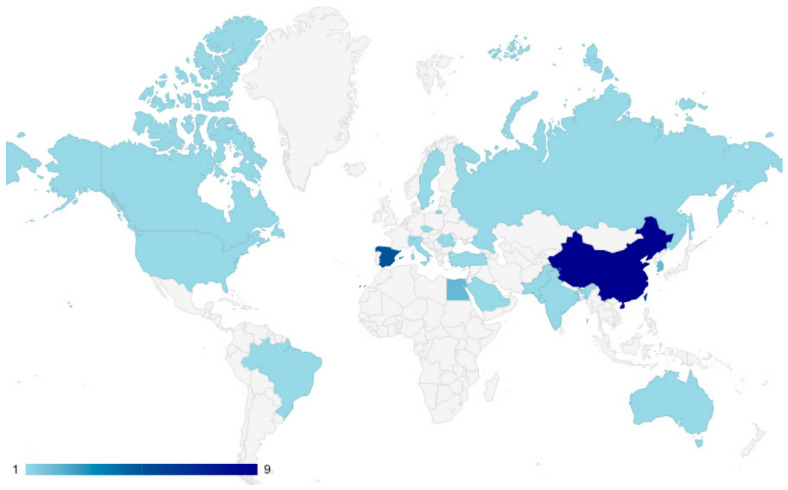
Global heatmap showing the geographical distribution of included trials in VR-based upper-limb stroke rehabilitation. The color intensity represents the number of studies contributed by each country (range: 1–9), with darker shades indicating higher study representation.

The geographical distribution of included trials is illustrated in Figure 2, underscoring the prevalence of VR-based rehabilitation studies from Asia (China, Taiwan, Korea) alongside additional contributions from Europe, North America and other countries involved in VR-based implementation protocols in upper-limb recovery post-stroke.

Most trials used some form of blinding: 25 (61.0%) were single-blinded (typically assessor-blinded), six (14.6%) were double-blinded, while 10 (24.4%) were unblinded.

Allocation was most frequently performed using computer-generated random sequences (*n =* 16), permuted block methods (*n =* 10), or sealed opaque envelopes (*n =* 13). Less frequent approaches included stratified randomization (*n =* 2), adaptive methods (*n =* 1), ballot selection (*n =* 1), and simple randomization without blocking (*n =* 3).

The dominant allocation ratio was 1:1 (*n =* 26), followed by 1:1:1 (*n =* 4) for three-arm designs. Unequal ratios were used in some trials (1:2, *n =* 5; 1:3, *n =* 1).

Table 3 defines the parameters of clinical trials and sample size, underscoring the variation in enrolled participants, completion rates and dropout rates between included trials.

### 3.3. Participant Characteristics

The reported age of participants ranged from 18 to 96 years. The earliest intervention was initiated within 3 days of stroke onset, whereas the most delayed case occurred up to 420 months (35 years) post-stroke. Table 4 summarizes participant demographic data and stroke characteristics, enabling an easy assessment of baseline features between EG and CG.

### 3.4. Study Populations

Across the included studies, eligibility criteria converged on several key domains, emphasizing time since stroke, age, motor-function thresholds, and cognitive–perceptual capacity. Nearly half of the trials enrolled participants in the acute to subacute phase [17,23,24,25,26,27,28,29,30,31,32,33,34,35,36,37,38,39,40,41,42,43,44], with some initiating interventions as early as the first week [32,38]. A smaller but notable proportion targeted chronic stroke survivors (≥6 months post-event) [19,45,46,47,48,49,50,51,52,53,54,55,56,57,58], with a few studies including individuals decades after stroke [31,49,56]. Only a minority adopted broad inclusion windows spanning multiple recovery stages [27,28,43,44,57,58]. All trials were limited to adult populations [≥18 years] [17,19,23,24,25,27,28,29,30,31,32,33,34,35,36,37,38,39,40,41,42,43,44,46,47,48,49,50,51,52,53,54,55,56,57,58,59,60], most commonly aged 45–70 years, though upper limits occasionally extended to 85–96 years [28,32,42,44].

Enrollment generally required a minimum level of voluntary movement in the affected upper limb, such as the ability to extend the wrist or fingers by 10–20° [41], lift the arm against gravity, or achieve Brunnström stage II–IV [19,23,25,27,30,40,42,43]. Most protocols targeted moderate to severe paresis, commonly operationalized as FMA-UE scores between 13 and 16 and 60 [23,31,34,37,43,60], Functional Independence Measure (FIM) scores ≥ 73 [34], or Modified Ashworth Scale spasticity grades < 4 [17,23,36,42,50]. Cognitive inclusion thresholds were consistently high, with most studies requiring Mini–Mental State Examination [MMSE] scores ≥ 21–24 [30,33,35,36,38,39,45,55,58] or Montreal Cognitive Assessment [MoCA] scores ≥ 15 [23,27,40], along with the ability to follow multi-step commands and the absence of severe aphasia [47]. Adequate visual function (e.g., acuity ≥ 20/50)and the absence of visual field deficits, such as hemianopia, were also standard [17,29,33,47,55].

Exclusion criteria demonstrated considerable overlap, reflecting shared concerns for safety, adherence, and feasibility. Participants with unstable systemic disease or cardiopulmonary instability (advanced heart failure, recent myocardial infarction, major hepatic/renal failure, pneumonia, severe anemia, arrhythmias, uncontrolled hypertension, advanced lung disease) were consistently excluded [19,23,27,29,33,40,44,48,50,52,53,59].

Neurological exclusions included recurrent or bilateral stroke, brainstem/cerebellar infarction, aneurysms, arteriovenous malformations, and progressive neurodegenerative disorders [32,35,39,42,46,53,57]. Contraindications also covered recent seizures, epilepsy, and recent botulinum toxin use or tone-modifying drugs [19,23,28,30,37]. Patients with coexisting neurological disease (traumatic brain injury, Parkinson’s, Alzheimer’s), severe cognitive impairment, dementia, or major psychiatric illness were not eligible [17,19,23,24,31,32,34,37,44,46,47,49,51,52,53,56,58].

Language impairments interfering with instructions, hemispatial neglect or apraxia were additional common exclusions [17,36,39,41,43,57]. Trials also ruled out participants with significant sensory or sensory–motor deficits [hemianopia, severe visual or hearing loss, vestibular dysfunction, anesthesia of the affected hand] [19,27,29,32,34,35,37,38,39,44,50,51,52,54,55,56,57,60,61]. Orthopedic and musculoskeletal issues—such as contractures, deformities, fractures, open wounds, severe shoulder pain, burns, or skin lesions—were also exclusionary [17,24,29,33,46,48,56,60]. Technical contraindications included metallic implants, pacemakers, cochlear implants, or the inability to undergo magnetic resonance imaging (MRI) [23,40,46,52]. Finally, studies excluded individuals with pregnancy, low anticipated compliance [refusal to consent, low attendance probability], concurrent trial enrollment, or inability to operate VR equipment [23,29,34,38,51].

### 3.5. Characteristics of the Intervention

#### 3.5.1. VR Type

A wide range of VR devices and protocols were used across the included trials, highlighting important heterogeneity. Non-immersive systems were most frequently applied for upper-limb recovery [25,27,28,29,30,32,33,34,35,36,40,41,43,44,45,46,47,48,50,52,53,55,56,58]. Immersive VR was used in 15 trials, and several of them explicitly reported head-mounted displays in their methodology [17,19,23,24,31,37,38,42,46,49,51,54,57,59]. Hybrid devices, including robotic or exoskeleton-assisted VR systems, were less commonly used [27,29,30,36,40,43,46].

**Figure 3:**
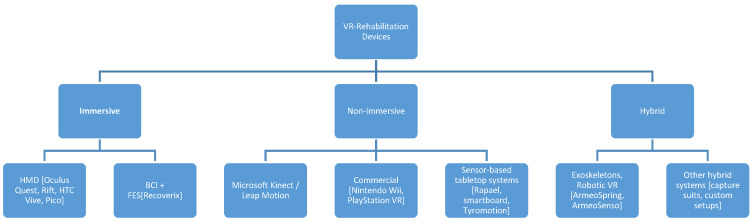
A schematic overview of VR devices across enrolled trials in our analysis. Abbreviations: Virtual reality (VR), head-mounted display (HMD), brain–computer interface (BCI).

VR- devices used in upper-limb rehabilitation in stroke patients can be classified into three main categories based on their immersion level and additional system integration. Figure 3 is a schematic overview of VR devices across the included clinical trials. Immersive systems such as head-mounted display (HMD) and brain–computer interface (BCI) platforms provide immersive environments that engage patients in interactive tasks and real time feedback recovery [25,27,28,29,30,32,33,34,35,36,40,41,43,44,45,46,47,48,50,52,53,55,56,58], while non-immersive systems use external sensors or screens to simulate movements and include devices like Microsoft Kinect or commercial gaming consoles and sensor-based tabletop platforms [17,19,23,24,31,37,38,42,46,49,51,54,57,59]. Hybrid systems or robotic systems combine VR devices with advanced motion captures, including exoskeletons [Armeo Spring, ArmeoSenso], designed to facilitate intensive recovery protocols and highly controlled rehabilitation [27,29,30,36,40,43,46]. VR-based systems used in upper-limb rehabilitation underscore the high diversity of proposed technologies and their recovery potentials to address different levels of motor impairment and the opportunity to define individualized rehabilitation protocols.

#### 3.5.2. VR Exposure/Duration

Across the included trials, VR session durations ranged from 15 to 60 min, with a mean of approximately 37 min (SD ≈ 15 min). Session frequency was clustered between two and five sessions per week, with most protocols prescribing either three or five sessions (mean 3 ± 1/week), indicating a high and consistent intervention intensity. Intervention periods varied substantially, from less than one week (0.75 week) to 12 weeks, with most programs concentrated in the 4–6 week range. The median duration was 4 weeks, lower than the mean of approximately 5.4 weeks, reflecting a right-skewed distribution driven by several longer protocols (10–12 weeks).

### 3.6. Patients’ Compliance and Adherence to VR Rehabilitation Protocols

**Figure 4:**
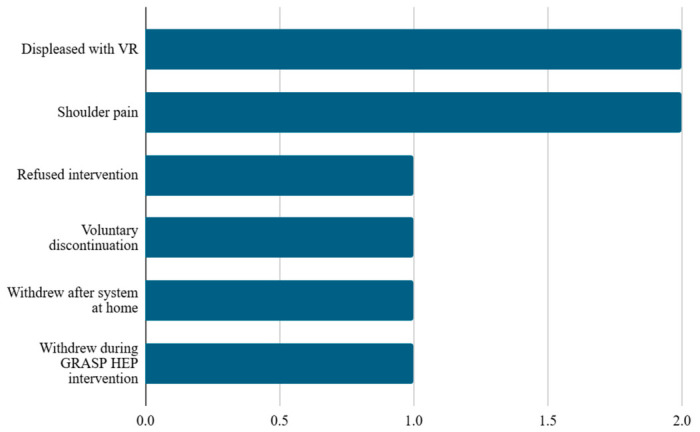
Reasons for dropout in the study related to virtual reality (VR) use. Abbreviations: Virtual Glove Rehabilitation Application for Stroke Patients Home Exercise Program (GRASP HEP) [27,28,31,40,56].

Across included studies, participant attrition was attributable to a wide range of medical, logistical, and study-specific factors. The most frequently reported medical causes for dropout included death [23], recurrent stroke [23], cardiovascular complications [e.g., atrial fibrillation, uncontrolled hypertension] [25,46,48], systemic comorbidities [anemia, diabetes, intercurrent illness] [25,40,46,48,52], and musculoskeletal injuries such as upper limb or hip fracture [46,49]. Non-medical factors encompassed withdrawal of consent, loss to follow-up, refusal to participate in follow-up assessments, transportation difficulties, transfer to another hospital, discharge from rehabilitation prior to study completion and relocation [28,40,43,46,56,58]. Specific reasons for participant dropout related to virtual reality (VR) use are illustrated in Figure 4.

### 3.7. Outcomes in Rehabilitation After Stroke

#### 3.7.1. Primary Outcomes

Across the reviewed stroke and neurorehabilitation trials, the FMA-UE was the most frequently utilized outcome measure, appearing in 70.7% of the included trials. Functional assessments such as the Action Research Arm Test (ARAT) (14.6%), Manual Function Test (MFT) (7.3%), and Wolf Motor Function Test (WMFT) (7.3%) were less frequent, serving as complementary measures of activity-level performance. Dexterity measures like the Box and Block Test (BBT) (7.3%) and spasticity measures like the Modified Ashworth Scale (MAS) (7.3%) were also used. Patient-reported and participation-level tools (e.g., Disabilities of the Arm, Shoulder and Hand or DASH, Stroke Impact Scale or SIS, Canadian Occupational Performance Measure or COPM) were comparatively rare (≤2.4%), as were neurophysiological markers (EEG/ERP, EMG). Of the 41 included studies, three were identified as having a ‘Critical Risk of Bias’ according to the ROBINS-I algorithm [29,35,49]. These specific trials utilized FMA-UE, EEG/EMG, and ARAT as their primary outcomes. The secondary outcomes in the three ‘Critical Risk’ trials included the BI, muscle-specific activation, BBT, and ABILHAND questionnaire.

#### 3.7.2. Secondary Outcomes

**Figure 5:**
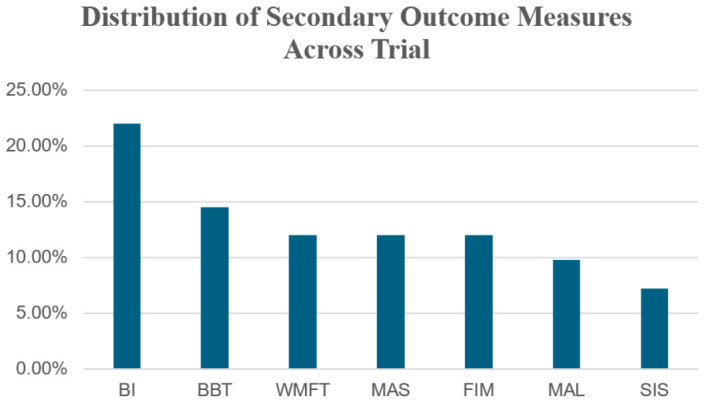
Descriptive frequency distribution of secondary outcome measures across included trials. Abbreviations: Barthel Index (BI), Box and Block Test (BBT), Wolf Motor Function Test (WMFT), Modified Ashworth Scale (MAS), Functional Independence Measure (FIM), Motor Activity Log (MAL), Stroke Impact Scale (SIS).

In the reviewed trials, secondary outcome measures reflected a multidimensional approach to post-stroke rehabilitation, encompassing impairment, activity, participation, and patient-centered domains. Figure 5 summarizes the descriptive frequency distribution of these various tools based on their frequency of use across the included studies. The Barthel Index (BI) was the most frequently utilized secondary measure, appearing in 22.0% of studies. This was followed by the BBT at 14.5%. Other assessments, including the WMFT, MAS, and FIM, showed a consistent usage rate of 12.0% each, while the MAL (9.8%) and SIS (7.2%) were used less frequently.

Less frequent but noteworthy were cognitive and sensory assessments (e.g., MoCA, two-point discrimination), strength and range of motion measures (e.g., Grip Strength, Active Range of Motion or AROM), and global disability indices (mRS, NIHSS). Rarely used metrics, such as neuropsychological tests, serum biomarkers, and adherence measures, indicate emerging but underutilized domains of evaluation.

### 3.8. Risk of Bias

The risk of bias for the included randomized controlled trials [17,19,23,24,25,27,28,30,31,32,33,34,36,37,38,39,40,41,42,43,44,45,46,47,48,50,51,52,53,54,55,56,57,58,59,60] was assessed using the revised Cochrane risk of bias tool (RoB 2). The assessment was performed independently by one reviewer, following Cochrane recommendations for domain-based judgment. The evaluation considered five domains.

**Figure 6:**
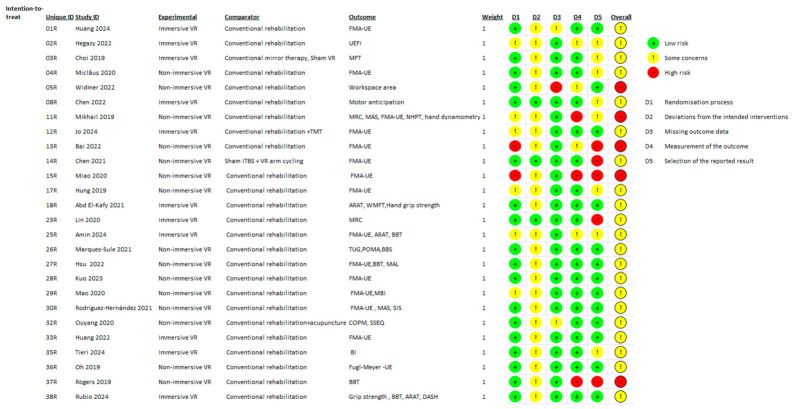
Risk of bias assessment (intention-to-treat analysis) [17,19,23,24,25,27,30,32,33,34,38,39,41,42,43,44,45,46,47,50,52,53,54,55,57,59,60]. Abbreviations: VR = Virtual Reality; FMA-UE = Fugl-Meyer Assessment for Upper Extremity; UEFI = Upper Extremity Functional Index; MFT = Manual Function Test; ARAT = Action Research Arm Test; BBT = Box and Block Test; WMFT = Wolf Motor Function Test; NHPT = Nine-Hole Peg Test; MAS = Modified Ashworth Scale; MRC = Medical Research Council Scale; TMT = Mirror Therapy; iTBS = Intermittent Theta-Burst Stimulation; TUG = Timed Up and Go Test; POMA = Performance-Oriented Mobility Assessment; BBS = Berg Balance Scale; MAL = Motor Activity Log; MBI/BI = [Modified] Barthel Index; SIS = Stroke Impact Scale; COPM = Canadian Occupational Performance Measure; SSEQ = Stroke Self-Efficacy Questionnaire; DASH = Disabilities of the Arm, Shoulder, and Hand.

**Figure 7:**
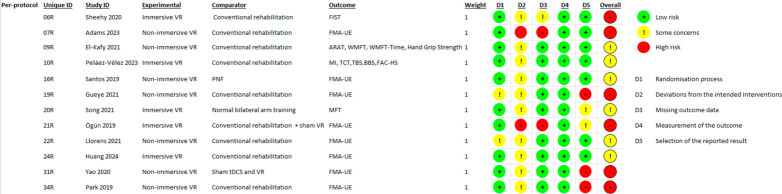
Risk of bias assessment (per-protocol analysis) [28,31,36,37,40,46,48,51,52,56,58,59]. Abbreviations: VR = Virtual Reality; FMA-UE = Fugl-Meyer Assessment for Upper Extremity; FIST = Function in Sitting Test; ARAT = Action Research Arm Test; WMFT = Wolf Motor Function Test; WMFT-Time = timed Wolf Motor Function Test; MI = Motricity Index; MFT = Manual Function Test; PNF = Proprioceptive Neuromuscular Facilitation; tDCS = Transcranial Direct Current Stimulation; TCT = Trunk Control Test; TBS = Tinetti Balance Scale; BBS = Berg Balance Scale; FAC-HS = Functional Ambulation Classification of Hospital of Sagunto.

Each study was rated as having low risk of bias, some concerns, or high risk of bias for each domain and overall. The visual summary of judgments for both the intention-to-treat and per-protocol analyses is presented in Figure 6 and Figure 7.

Among the non-randomized studies [29,35,49] assessed with ROBINS-I, three were rated as having a critical risk of bias in Domain 1 (confounding). In accordance with the ROBINS-I algorithm, these studies were not evaluated in subsequent domains, and their overall risk of bias was deemed critical. The remaining non-randomized studies were assessed across all domains, generally showing moderate to serious risk due to issues with intervention classification and missing data.

## 4. Discussion

### 4.1. Primary Outcomes

The heavy reliance on the FMA-UE (70.7%) confirms its status as the “gold standard” for measuring motor impairment [62], yet its dominance reveals a significant gap in stroke research. The rarity of patient-reported outcomes (e.g., SIS, COPM at ≤2.4%) indicates that the survivor’s personal experience and social participation remain secondary to clinical observation. The lack of neurophysiological markers (EEG/EMG) may hinder our ability to distinguish between true neural recovery and simple behavioral compensation.

### 4.2. Secondary Outcomes and Holistic Rehabilitation

While motor recovery remained the dominant focus, secondary outcomes revealed VR’s broader rehabilitative potential. Several trials reported improvements in functional independence (e.g., FIM), dexterity (Box and Block Test, ARAT), and spasticity reduction (Modified Ashworth Scale). These findings emphasize that VR interventions can extend beyond impairment-level changes to support activity and participation, which are critical for reintegration into daily life. Cognitive domains, mood, and QoL were less frequently assessed but consistently showed promising trends, including enhanced motivation and reduced depressive symptoms in select interventions [14,63]. Importantly, these outcomes highlight areas where conventional rehabilitation often falls short, suggesting that VR-based approaches may uniquely address the psychosocial and cognitive sequelae of stroke.

However, the heterogeneity in secondary outcome measures—ranging from disease-specific scales to generic questionnaires—limits interpretability and cross-trial comparability. The scarcity of patient-reported outcomes and biomarkers further represents a missed opportunity to capture VR’s full impact on holistic recovery and neuroplasticity. Emerging use of neurophysiological measures, such as EEG and EMG, underscores the potential for mechanistic insights but remains underutilized. Collectively, this variability underscores both the promise and the current limitations of the evidence base [26]. To advance the field, future trials should systematically integrate standardized, multidomain outcome frameworks that encompass motor, functional, cognitive, emotional, and quality-of-life dimensions [14,64]. Such harmonization would enable more robust synthesis of evidence and ensure that VR-based rehabilitation protocols are evaluated against the full spectrum of challenges faced by stroke survivors.

### 4.3. Motivation, Compliance, and Safety

A key differentiator between VR and conventional therapy lies in engagement. VR systems provided immersive, gamified, and feedback-driven environments that promoted motivation and adherence [65], with mean dropout rates of only 3% across trials. Discontinuations were rarely attributable to VR itself; the few cases reported were primarily related to shoulder pain or personal choice to discontinue. By contrast, adherence in conventional therapy is often undermined by monotony and resource limitations [22]. The consistently low attrition rates in VR trials support both the safety and the economic feasibility of integrating VR into clinical practice and home-based rehabilitation programs.

### 4.4. Accessibility and Equity of Care

A critical implication of VR-based rehabilitation lies in its capacity to overcome traditional barriers of access. Conventional therapy is resource-intensive, often requiring specialized facilities and therapist availability, which can be limited in low-resource settings. VR-based platforms—particularly non-immersive or portable systems—enable rehabilitation to be delivered at home under remote supervision [66]. This not only reduces the burden on healthcare infrastructure but also empowers patients and caregivers to actively participate in recovery. However, equitable implementation requires addressing cost disparities, digital literacy, and access to reliable internet, which remain unevenly distributed across healthcare systems [1,14,67].

### 4.5. Device Heterogeneity and Implementation Challenges

The reviewed trials employed a heterogeneous spectrum of VR devices, ranging from non-immersive screen-based systems (e.g., Kinect, tabletop sensors) to immersive head-mounted displays and robotic or exoskeleton-assisted hybrids (e.g., Armeo Spring, ArmeoSenso). While immersive and hybrid systems often reported greater motor gains—particularly when paired with high-intensity, task-oriented training—this diversity complicates direct comparisons and hinders the development of standardized clinical guidelines [68,69]. Importantly, device heterogeneity reflects both rapid technological advancement and the potential for individualized rehabilitation pathways. For example, immersive systems may maximize engagement in younger or cognitively intact patients, whereas simpler non-immersive platforms could improve feasibility in older adults or resource-limited settings [70,71]. At the same time, cost, usability, and accessibility remain critical barriers to large-scale adoption, particularly in health systems with limited rehabilitation resources [14,71]. Future multicenter trials should stratify outcomes by device type, task characteristics, and stroke severity to identify optimal matches between technology and patient profile. Ultimately, VR should be conceptualized as an adaptable adjunct to conventional therapy, with harmonized protocols that balance standardization for comparability and flexibility for personalized care.

### 4.6. Monitoring and Personalized Feedback

VR interventions provide unique opportunities for objective, continuous monitoring of motor performance. Unlike conventional rehabilitation, which often relies on episodic therapist assessments, VR systems can generate real-time kinematic and performance data, enabling precise tracking of recovery trajectories [69]. This data-rich environment supports personalized feedback, adaptive task progression, and early detection of plateaus or regressions [72]. Integration with artificial intelligence could further enhance predictive modeling, tailoring rehabilitation intensity and content to the patient’s stage of recovery and impairment severity [33].

### 4.7. Limitations

This review has several limitations that warrant careful consideration. First, although the vast majority of included trials were randomized, sample sizes remained modest, with a median enrollment of fewer than 40 participants. Such underpowered designs restrict the precision of effect estimates and limit the possibility of subgroup analyses by stroke stage, impairment severity, or device type. Second, the heterogeneity of interventions—spanning multiple VR devices, task designs, treatment durations, and intensity regimens—precluded a quantitative meta-analysis. While this heterogeneity reflects ongoing technological innovation, it complicates the synthesis of results and underscores the urgent need for harmonized reporting standards.

A notable limitation of this review is the restricted timeframe (2019–2024). While this may have excluded earlier foundational trials, the rapid pace of technological innovation in VR rendering and sensor precision makes older data less applicable to modern clinical implementation. Furthermore, previous meta-analyses covering earlier periods have already established the base efficacy of VR; thus, this review serves as a necessary temporal update focusing on the latest generation of VR interventions.

Most RCTs assessed with RoB 2 showed low to moderate risk of bias, with the main concerns related to lack of participant or therapist blinding and unclear adherence to pre-specified analysis plans. Randomization methods were generally adequate, and outcome assessors were often blinded, supporting overall reliability. In contrast, the non-randomized studies assessed with ROBINS-I showed higher risks, mainly due to confounding and participant selection.

Strengthening trial design, preregistration, and blinding procedures is essential for future research.

The scarcity of long-term follow-up further constrains conclusions regarding the durability of treatment effects.

### 4.8. Future Directions

Taken together, these limitations highlight the necessity for large-scale, multicenter RCTs that employ standardized VR protocols, core outcome sets, and transparent reporting. Only through such methodological refinement can the field progress toward defining best-practice frameworks and optimizing the clinical integration of VR-based rehabilitation in stroke care.

The evidence base is dominated by small single-center RCTs, with a mean sample size of 41 participants and limited representation outside Asia and Southern Europe. Larger, multicenter RCTs with standardized VR protocols and cross-culturally validated outcomes are urgently needed to strengthen external validity. Future research should also integrate long-term follow-up to establish the durability of effects and cost-effectiveness analyses to support scalability.

To address the limited depth arising from substantial inter-study heterogeneity, future research should move toward a stratified framework that clearly defines the conditions under which VR interventions are most effective. One of the most critical factors is the timing of delivery, as the subacute phase represents a relatively brief yet highly sensitive window in which recovery potential is greatest. Accordingly, studies should distinguish between outcomes achieved during early restorative processes and those reflecting later compensatory adaptations. Research should explicitly compare levels of immersion, recognizing that fully immersive systems may provide richer sensory feedback and greater motivational rewards than screen-based interventions. Determining the minimum effective dose necessary to promote neural reorganization is essential for establishing evidence-based guidelines that optimize therapeutic impact and support sustained functional recovery.

Technological advances offer further opportunities. Telerehabilitation platforms could extend access to underserved populations, while AI-based monitoring, real-time feedback, and biomarker-driven personalization may enhance adherence and optimize therapy intensity. Combining VR with complementary modalities such as wearable sensors, BCIs, and neurostimulation could yield synergistic effects by simultaneously targeting motor learning, neuroplasticity, and functional independence.

## 5. Conclusions

By synthesizing 41 recent trials published between 2019 and 2024, this review provides a comprehensive map of the current VR landscape in stroke neurorehabilitation. While the FMA-UE remains the most utilized scale for assessing motor recovery, our analysis reveals a significant trend toward multidimensional evaluation. The study identifies critical gaps in standardization and scalability, and highlights future avenues for multimodal, personalized neurorehabilitation. Several critical gaps remain that hinder the large-scale clinical adoption of VR. Variations in the clinical window of intervention, coupled with inconsistent prescriptions of intensity (total hours and session frequency), remain major barriers. The review found high levels of treatment tolerability; dropout rates remained low, with most withdrawals occurring for reasons external to the study protocols. Furthermore, the low incidence of adverse events suggests that VR is a safe and feasible modality for clinical integration, provided that patient-specific clinical features are considered during implementation.

## Figures and Tables

**Table 1:** Challenges in conventional rehabilitation protocols vs. potential benefits of VR-based interventions for stroke recovery.

Domain	Conventional Rehabilitation Protocols	VR-Based Rehabilitation Potential Benefits	Clinical Impact in Stroke Rehabilitation
Motivation	Decreased motivation for prolonged stroke rehabilitation protocols	Gamification, real-time feedback, goal-oriented task	Increased intensity and sustained long-term motivation [14,15]
Engagement	Low commitment due to repetitive, monotonous exercises	Immersive and interactive environments significantly increase commitment and participation	Promotes neuroplasticity through active and sustained practice in home-based and clinical settings [16,17]
Adherence	Decreased adherence and high dropout rates due to prolonged protocols and limited accessibility	Remote access, personalized tasks, and real-time feedback improve protocol adherence	Supports long-term rehabilitation adherence and continuity of care [18,19]
Accesibility	Limited by physical therapist availability, cost, and travel to rehabilitation centers	Home-based protocols with minimal supervision provide broader accessibility	Extends rehabilitation reach beyond traditional clinical settings [9,19]
Monitoring and standardization	Variable therapist protocols; challenges in accurately tracking rehabilitation progress	Tele-monitoring capabilities; digital tracking of performance and recovery metrics	Enables personalized protocol adjustments based on objective, data-driven evaluation of progress [20,21,22]

**Table 2:** Eligibility criteria for study inclusion.

Domain	Criteria
Population	Adult stroke survivors (≥18 years), ischemic or hemorrhagic stroke, at acute, subacute, or chronic stage; time since onset explicitly reported.
Intervention	VR–based rehabilitation protocols specifically targeting upper-limb motor and/or functional recovery, delivered in immersive or non-immersive formats.
Comparator	Conventional therapy, sham intervention, or alternative non-VR rehabilitation intervention when applicable; comparators were not required for non-randomized designs.
Outcomes	At least one objective upper-limb motor or functional outcome (e.g., FMA-UE) or equivalent validated measure. Secondary outcomes could include dexterity, motor function, spasticity, functional independence, cognition, mood, and QoL
Study Design	RCTs and non-randomized designs, including observational studies, single-case, and cross-sectional designs.
Timeframe	Studies published between January 2019 and December 2024.
Language restriction	English

**Table 3:** Enrollment, completion and dropout data across trials.

Metric	Enrolled Participants	Completed (No Follow-Up)	Completed (+ Follow-Up)	Dropout Rate (No Follow-Up)	Dropout Rate (+ Follow-Up)
Mean	40.9	39.5	38.1	3%	3%
Median	37	36	36	0%	0%
Min	5	5	5	0%	0%
Max	152	145	143	29%	17%
Sum	1676	1539	1411	–	–

**Table 4:** Baseline demographic and clinical characteristics.

Variable	Experimental Group (EG)	Control Group (CG)	Total
Number of participants	800	830	1470
Male, n (%)	472 (59.0%)	458 (55.2%)	930 (63.3%)
Female, n (%)	252 (31.5%)	288 (34.7%)	540 (36.7%)
Mean Age ± SD (years)	58.2 ± 11.3	59.3 ± 10.7	–
Time since stroke	–	–	–
<6 months (n, %)	21 (52.5%)	17 (47.2%)	–
≥6 months (n, %)	17 (42.5%)	16 (44.4%)	–
Mixed phases (n, %)	2 (5.0%)	3 (8.3%)	–

## Data Availability

No new data were generated for this systematic review. All data supporting the findings are available within the cited studies.

## Supplementary Materials

The following supporting information can be downloaded at the following: S1. PROSPERO: https://www.crd.york.ac.uk/PROSPERO/view/CRD420251150877 (all accessed on 13 April 2026); S2. Search string: https://docs.google.com/document/d/1o-oPx_2hq46vVS_bUoTn-oVJ-Lhpc-1P/edit?usp=drive_link&ouid=110050764088942135689&rtpof=true&sd=true; Table S1. PRISMA 2020 Checklist: https://docs.google.com/document/d/1ilcFEnb5I7eN-sUacuvWfilRJIRd-hNo/edit?usp=sharing&ouid=110050764088942135689&rtpof=true&sd=true; Table S2. PRISMA 2020 for Abstracts checklist: https://docs.google.com/document/d/1a7x259WQVtNy6TOMdF2UHDPnvzIGiWw3/edit?usp=sharing&ouid=110050764088942135689&rtpof=true&sd=true; Table S3. Titles of studies excluded after full-text review: https://docs.google.com/spreadsheets/d/1JRaVZCT7pYAbKpmZtXBjc6IKka8pFa0w/edit?usp=drive_link&ouid=110050764088942135689&rtpof=true&sd=true; Table S4. Summary of the main results of all included studies: https://docs.google.com/spreadsheets/d/1ZhKbftvcVx-N2yL0SYGd3sSh6k2i9sXE/edit?usp=drive_link&ouid=110050764088942135689&rtpof=true&sd=true. Reference [73] is cited in the supplementary materials.

## Abbreviations

The following abbreviations are used in this manuscript:

ARAT	Action Research Arm Test
AROM	Active Range of Motion
BBT	Box and Block Test
BBS	Berg Balance Scale
BCI	Brain–Computer Interface
BI/MBI	(Modified) Barthel Index
CENTRAL	Cochrane Central Register of Controlled Trials
CG	Control Group
COPM	Canadian Occupational Performance Measure
DASH	Disabilities of the Arm, Shoulder and Hand
EEG/ERP	Electroencephalography/Event-Related Potentials
EG	Experimental Group
EMG	Electromyography
FAC	Functional Ambulation Classification
FIM	Functional Independence Measure
FIST	Function in Sitting Test
FMA-UE/FMUE	Fugl–Meyer Assessment—Upper Extremity
GRASP HEP	Glove Rehabilitation Application for Stroke Patients—Home Exercise Program
HMD	Head-Mounted Display
iTBS	Intermittent Theta-Burst Stimulation
MAL	Motor Activity Log
MAS	Modified Ashworth Scale
MCID	Minimal Clinically Important Difference
MFT	Manual Function Test
MI	Motricity Index
MMSE	Mini–Mental State Examination
MoCA	Montreal Cognitive Assessment
MRC	Medical Research Council Scale
MRI	Magnetic Resonance Imaging
NHPT	Nine-Hole Peg Test
PNF	Proprioceptive Neuromuscular Facilitation
PROSPERO	International Prospective Register of Systematic Reviews
QoL	Quality of Life
RCTs	Randomized Controlled Trials
RoB 2	Risk of Bias 2 Tool
ROBINS-I	Risk of Bias in Non-Randomized Studies—of Interventions
SIS	Stroke Impact Scale
SR	Systematic Review
SSEQ	Stroke Self-Efficacy Questionnaire
TBS	Tinetti Balance Scale
TLA	Three-Letter Acronym
TMT	Mirror Therapy
tDCS	Transcranial Direct Current Stimulation
TUG	Timed Up and Go Test
UEFI	Upper Extremity Functional Index
VR	Virtual Reality
WMFT/WMFT-Time	Wolf Motor Function Test/Timed WMFT
